# YKL-40-Loaded Amniotic Fluid Exosomes Promoted Angiogenesis Response in a Rat Model of Acute Myocardial Infarction

**DOI:** 10.34172/apb.46571

**Published:** 2026-05-25

**Authors:** Hamid Lotfimehr, Narges Mardi, Reza Rahbarghazi, Mohammad Nori, Shahram Rabbani, Hamid Tayefi Nasrabadi

**Affiliations:** ^1^Stem Cell Research Center, Tabriz University of Medical Sciences, Tabriz, Iran; ^2^Department of Applied Cell Sciences, Faculty of Advanced Medical Sciences, Tabriz University of Medical Sciences, Tabriz, Iran; ^3^Student Research Center, Tabriz University of Medical Sciences, Tabriz, Iran; ^4^Research Center for Advanced Technologies In Cardiovascular Medicine, Cardiovascular Diseases Research Institute, Tehran University of Medical Sciences, Tehran, Iran; ^5^Stem Cell and Regenerative Medicine Institute (SCARM), Tabriz University of Medical Sciences, Tabriz, Iran

**Keywords:** Angiogenesis, Fibrosis, Myocardial infarction, Regeneration, YKL-40 peptide

## Abstract

**Purpose:**

Blood re-establishment into ischemic myocardium can salvage cardiomyocytes from pathologic cardiac remodeling. Extracellular vesicles (EVs), especially exosomes (Exos), have been used as suitable nanocarriers for the delivery of cytokines to different tissues. Here, the therapeutic properties of YKL-40-loaded amniotic fluid Exos (YKL-40@AFExos) were assessed in infarcted rats.

**Methods:**

Human AFExos were loaded with exogenous YKL-40 via sonication, and YKL-40@AFExos were injected directly into the border zone of ischemic myocardium in rats. After two weeks, rats were euthanized, and the angiogenesis properties and fibrotic changes were studied using histological examination and western blotting.

**Results:**

Data indicated loading efficiency of YKL-40 onto AFExos was in the range of 55.5±1.5%. Masson’s trichrome staining revealed the reduction of collagen fibers in rats that received YKL-40@AFExos compared to MI and YKL-40 groups (*P*<0.05). These features coincided with the reduction of recruited immune cells and an increase in viable cardiomyocytes. IHC staining confirmed the significant increase of alpha-smooth muscle actin (α-SMA) and von Willebrand factor (vWF) vessels in YKL-40@AFExos, AFExos, and YKL-40 groups compared to the MI rats (*P*<0.05). Administration of YKL-40@AFExos in MI rats significantly reduced apoptosis (Bax↓) and autophagic response (LC3-II/I ratio↓) (*P*<0.05). The injection of YKL-40 alone can promote angiogenesis via the local increase in VEGFR-1, but not VEGFR-2 and VEGFR. These values did not reach significant levels in YKL-40@AFExos and AFExos groups.

**Conclusion:**

Transplantation of YKL-40@AFExos in infarcted rats led to regeneration of ischemic myocardium via enhancing angiogenesis and reduction of fibrotic changes, rather than YKL-40 peptide alone.

## Introduction

 Myocardial infarction (MI) is the main cause of human death, with high rates of morbidities and mortalities in developed and developing countries.^1^ In general, MI is induced by complete interruption or diminished blood perfusion into the heart ventricles due to the formation of clots or rupture of atherosclerotic plaques in the coronary artery.^2,3^ In MI patients, ST-elevation and non-ST-elevation MI patterns have been introduced.^4^ Although advances in therapeutic modalities and pharmacological regimes have led to a significant reduction in MI-related casualties, MI post-complications are at high levels.^5^ It has been reported that the occurrence of heart failure, an irregular heartbeat, cardiogenic shock or arrest, and impaired diastolic and systolic function are common in MI patients.^6,7^ Following MI and the loss of sarcolemma membrane integrity in injured cardiomyocytes, the release of specific cardiac tissue factors such as creatine kinase, troponin I, and T is stimulated, and their blood levels reach the maximum values after 24 hours. Along with these changes, irreversible changes such as cardiomyocyte swelling and cardiomyocytolysis are evident with prominent inflammation at the border zone of the necrotic area, resulting in fibrotic changes and scar formation after 6 weeks postinfarction.^8^

 Conventional therapies in MI patients mainly focus on the use of stepwise approaches to restore blood perfusion into the infarcted myocardium to prevent the extension of the necrotic area.^1,9^ The application of several drugs, such as antiplatelets, anticoagulants, nitrates, beta-blockers, statins, and surgical approaches [percutaneous coronary intervention (PCI), and coronary artery bypass graft (CABG)] is beneficial to alleviate MI-related pathologies.^10,11^ Unfortunately, these therapeutic strategies often did not completely restore the function of injured sites after a heart attack. Besides, in patients who underwent CABG or PCI, the risk of post-surgical side effects such as infection, arrhythmias, bleeding, thrombus formation, stroke, and renal tissue dysfunction should not be overlooked.^12-14^ Therefore, the development and advent of *de novo* therapeutic approaches are mandatory in MI cases to yield higher patient outcomes.

 In recent years, the application of various stem cells and their byproducts has increased for the alleviation of different ischemic conditions.^15^ Extracellular vesicles (EVs) with different subsets, including exosomes (Exos), microvesicles (MVs), and apoptotic bodies, are involved in donor cells’ to the recipient cells with the transfer of diverse signaling molecules.^16^ Among EVs, Exos with an average diameter size of 50-250 nm are valid bioshuttles for the loading and delivery of therapeutics into the target sites.^17^ It has been indicated that stem cells and EVs can promote the regeneration of infarcted myocardium after transplantation via the stimulation of angiogenesis and an increase in microvascular density.^18^ It has been shown that EVs with specific molecular signatures can orchestrate the angiogenesis potential via the regulation of different signaling pathways.^19^ Recent advances in biotechnology and nanotechnology approaches have led to smart manipulation of EVs to transfer the angiogenesis compounds for increasing therapeutic outcomes.^17,20^ The higher retention time, synergistic effects with encapsulated cargoes, and ability to transfer the natural barriers make EVs superior compared to direct factor or drug therapy.^21^ Besides, Exos with low immunogenicity and cytocompatibility are premium bioshuttles for factor delivery compared to the synthetic counterparts.^22^

 In recent decades, numerous proangiogenesis factors and subcellular units have been identified with distinct bioactivities, leading to an increase in our understanding of the blood formation mechanisms.^23,24^ Among them, YKL-40, known also as Chitinase-3-like protein 1, is a glycoprotein produced by inflammatory cells and tumor cells and can stimulate angiogenesis under physiological and pathological conditions via the regulation of endothelial cell (EC) activities, especially in tumor parenchyma.^25,26^ It seems that YKl-40 has synergy with the vascular endothelial growth factor (VEGF) signaling pathway, in which the suppression of this factor can blunt the vascularization via the VEGF/VEGF receptor-2 (VEGFR2) and ERK1/2 signaling axis.^27^ To the best of our knowledge, there are few reports related to the angiogenic properties of YKL-40 and YKL-40-loaded Exos in animal models of infarction. Whether the load of YKL-40 into Exos can enhance the angiogenesis properties of this factor has not been elucidated in previous works.

 In the current study, YKL-40-loaded amniotic fluid Exos (AFExos) were used as angiogenesis switchers in a rat model of MI to reduce the intensity of pathologies (Figure 1). Data from this study can help us understand the angiogenesis potency of YKL-40 under hypoxic/ischemic conditions, especially under MI circumstances.

**Figure 1:**
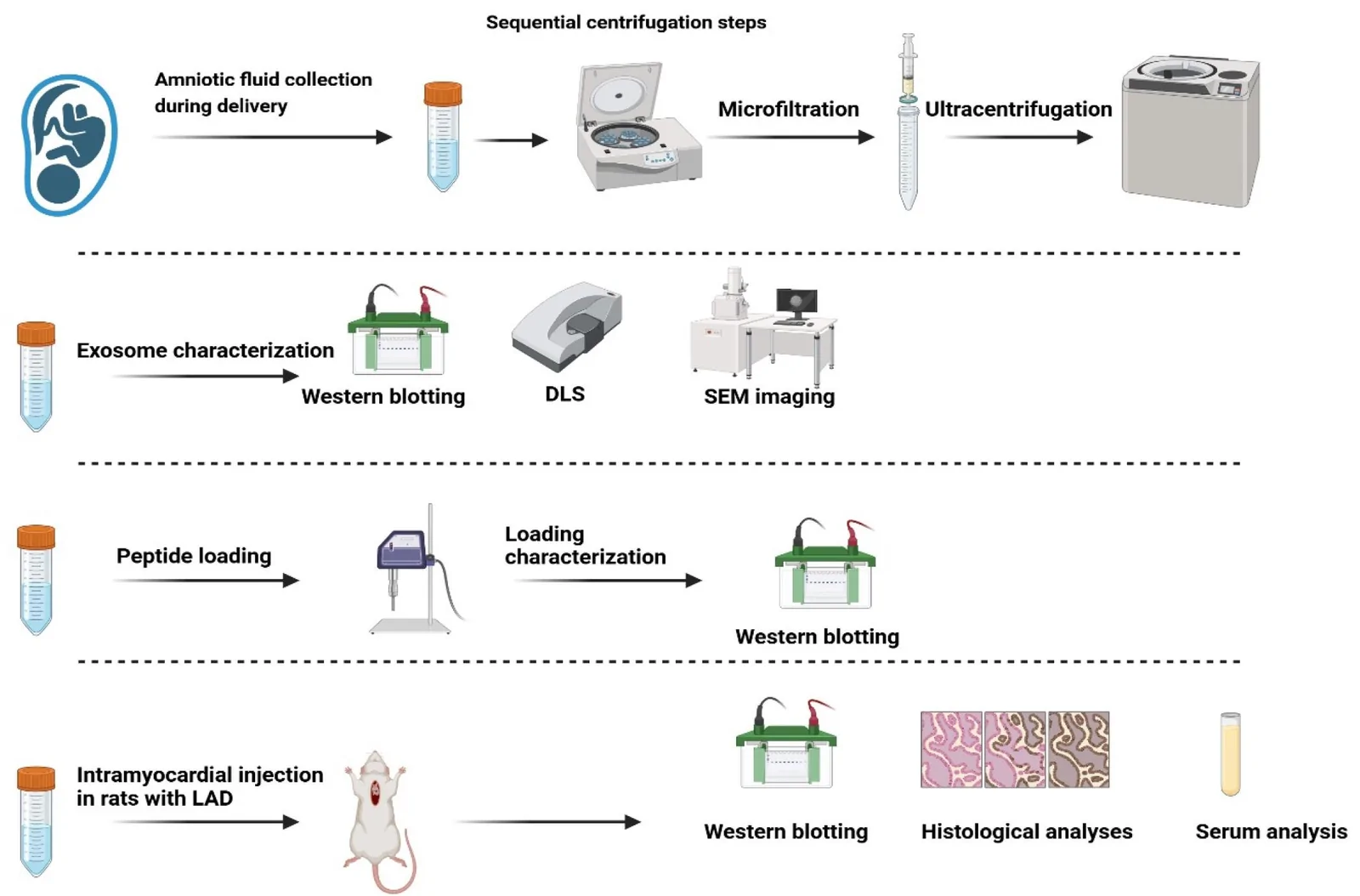
Schematic illustration of the current study. Physicochemical properties of isolated AFExos were confirmed using western blotting, scanning electron microscopy (SEM), and transmission electron microscopy (TEM) imaging. YKL-40 peptide was loaded onto the AFExos using sonication. The angiogenic properties of YKL-40@AFExos were evaluated two weeks after injection into the border zone of the ischemic area in a rat model. Designed by web-based BioRender

## Material and Methods

###  Animal Ethical Issues

 Thirty male Wistar rats, weighing ~180-200g, were purchased from Royan Institute (Tehran), and housed in the animal house of Tehran Heart Center under standard conditions (12 h/12h dark cycle, 20-22°C with 50-60% relative humidity. Animals were kept inside the standard cages with free access to water and chewing pellets. All steps of this study were approved by the Elite Researcher Grant Committee under award number [IR.NIMAD.REC.1397.512] from the National Institute for Medical Research Development (NIMAD), Tehran, Iran, and Tabriz University of Medical Sciences under the ethical code of IR.TBZMED.VCR.REC.1397.395. Animals were treated according to the previously published ARRIVE guidelines. Rats were randomly allocated to five groups (each in 6) as follows: Control, MI, AFExos, YKL-40, and YKL-40@AFExos groups. Before beginning an experimental procedure, rats were allowed to adapt to the conditions.

###  Isolation of AFExos

 AFExos were isolated from amniotic fluids subjected to amniocentesis in pregnant females between 15 and 20 weeks of pregnancy without influencing the diagnosis and any therapeutic protocols. The volunteers were asked to complete the informed consent forms. The samples were transferred to 50 ml sterile conical tubes to the cell culture laboratory of the Faculty of Advanced Medical Sciences. Samples were diluted at a ratio of 1: 1 with phosphate-buffered saline (PBS) and subjected to sequential centrifugation steps as follows: 300 g for 10 minutes; 2000 g for 15 minutes, and 10,000 g for 20 minutes. Then, the samples were passed using 0.22 μm microfilters, and exosomal pellets were harvested using ultracentrifugation at 100,000 for 60 minutes (Optima^TM^ TLX-120 ultracentrifuge, Beckman Coulter Inc. USA). All centrifugation steps were done at 4°C.

###  Characterization of AFExos

####  Scanning Electron Microscopy (SEM) and Transmission Electron Microscopy (TEM)

 The morphology and size of AFExos were monitored using SEM and TEM images according to the previous protocols.^16^ To this end, AFExos were diluted in distilled water, placed on aluminum foil, and allowed to air dry. Samples were fixed with 2.5% glutaraldehyde solution (Cat No. 354400, Sigma = Aldrich, USA) and gold sputtered. Images were taken using a Mira-3 FEG SEM microscope (Tescan Co. Brno, Czech Republic). The mean diameter of AFExos was measured using the ImageJ software version. 1.4. (NIH, USA). For TEM analysis, ~25 µl of diluted AFExo solutions were carefully transferred onto carbon-coated 300-mesh copper grids and stained with 2% wt./v uranyl acetate solution, followed by covering with carbon films and imaged using. TEM apparatus (LEO 906, Zeiss, Germany) under 100 kV.

####  Dynamic Light Scattering (DLS) 

 The hydrodynamic diameter of diluted AFExos was studied using the DLS device Malvern Nano ZS (Malvern Panalytical Ltd, UK).

####  Western Blotting

 Protein levels of tetraspanins such as CD63, TSG101, and CD81 were detected to confirm the presence of AFExos. Samples were lysed using protein lysis buffer (RIPA), and protein levels were detected using a BCA kit (Cat No: 21071, SMART^TM^ Micro BCA Protein Assay; iNtRON Biotechnology Inc., Gyeonggi-do, Republic of Korea). About 10 µg of exosomal protein was loaded in each lane of a 10% SDS-PAGE gel, and protein content was separated inside the electrical field using electrophoresis buffer. After transferring onto the PVDF membranes (Bio-Rad Laboratories; USA), and blocking with 1% bovine serum albumin (BSA, Cat No. A2153, Sigma Aldrich, MO, USA) for 30 minutes, anti-CD63 (Cat no: sc-5275; Santa Cruz Biotechnology Inc., USA), -TSG-101 (Cat no: sc-7964; Santa Cruz Biotechnology Inc., USA), and -CD81 (Cat no: sc-166029; Santa Cruz Biotechnology Inc., USA) and HRP-tagged secondary antibodies were used to detect the target proteins. Using ECL solution and X-ray films, immunoreactive bands were visualized, and densitometry analysis was done using ImageJ software (Ver. 1.4; NIH).

###  YKL-40 Peptide Loading Onto the AFExos

 In the current study, the YKL-40 peptide was loaded onto the isolated AFExos using the sonication method. For this purpose, 100 µg/ml of bioactive recombinant full-length YKL-40 protein (Cat no: ab182706; Purity > 95%; Abcam; USA) was incubated with AFExos corresponding to ~100 µg/ml exosomal protein. The samples were put inside the ice-cold water and subjected to the sonication protocol (Bandelin Sonopuls, Germany) with an amplitude setting of 20%, six cycles (bouts of 30 seconds each), and a 2-minute cooling interval between cycles. After the completion of sonication time, the samples were ultracentrifuged, and exosomal pellets were analyzed for the levels of loaded peptide using western blotting compared to peptide-free AFExos. In brief, the loaded and unloaded AFExo samples were lysed using protein lysis buffer, and YKL-40 levels were studied using anti-YKL-40 (Cat no: sc-393494; Santa Cruz Biotechnology Inc., USA) and HRP-tagged secondary antibodies. Protein levels of YKL-40 were measured using the BCA pre- and post-loading steps.

###  Induction of an Experimental MI Model in Rats

 To induce myocardial ischemia in rats, the left anterior descending artery (LAD) was ligated according to our previously published data.^28,29^ Rats were anesthetized using the combination of 50 mg/kg ketamine and 1 mg/kg medetomidine via i.m. and then connected to a ventilation system (Sulla 909 V, Dräger, Germany) with a gas chamber to inhale anesthetic agents composed of halothane with NO (50% v/v) and O_2 _(50% v/v), and CO_2_ (5% v/v). Left ventricles (LV) were exposed via left thoracotomy, and LAD was ligated using 5-0 silk suture strings. The infarcted myocardium turned pale about 20 minutes after LAD occlusion, with the elevation of ST segments in the electrocardiogram cycles. About 30 minutes post-LAD ligations, a total volume of 100 µl AFExo samples corresponding to 100 µg/ml exosomal protein were injected into four regions at the border zones of infarcted areas. In the YKL-40 group, rats received equal levels of YKL-40 peptide dissolved in 100 µl solvent similar to YKL-40@AFExo. The rats of the MI groups received 100 µl of vehicle. The exosomal protein content was equal in all AFExo-treated rats. After 14 days, rats were euthanized using an overdose of ketamine and xylazine. The process was continued by heart tissue sampling. For this end, hearts were carefully sliced transversely into 4-5 sections from base to apex and subjected to different analyses.

###  Western Blotting

 In slices 3, protein levels of different proteins related to angiogenesis (VEGF, VEGFR-1, and VEGFR-2), autophagy (LC3II/I ratio), and apoptosis (Bax and Bad) were monitored using western blotting. In short, samples were lysed using protein lysis buffer and subjected to 10% SDS-PAGE electrophoresis. The process was continued by transferring separated proteins onto the PVDF membranes and incubation with anti-VEGF (Cat no: E-AB-67255; Elabscience^®^, Texas, USA), -VEGFR-1 (Cat no: sc-271789; Santa Cruz Biotechnology, Inc., USA), -VEGFR-2 (Cat no: sc-6251; Santa Cruz Biotechnology, Inc., USA), -Bax (Cat no: sc-526; Santa Cruz Biotechnology, Inc., USA), -Bad (Cat no: sc-8044; Santa Cruz Biotechnology, Inc., USA), -LC3 (Cat no: #2775; Cell Signaling Technology, USA), and visualized using HRP-conjugated secondary antibodies (Cat no: sc-516102, and sc-2357; all purchased from Santa Cruz Biotechnology, Inc., USA), X-ray films and ECL solution. Each protein level was normalized to housekeeping β-actin protein (Cat no: sc-517582; Santa Cruz Biotechnology, Inc., USA).

###  Histological Examination

####  H & E and Masson’s Trichrome Staining

 In slice 4, histological examinations were done. Samples were fixed in 10% buffered formalin solution, and 5 μm-thick paraffin sections were prepared and stained with H & E solution. For the calculation of the infarcted area, 5 μm-thick slides were stained using Masson’s trichrome solution. The infarcted areas were calculated relative to the total area in each section using ImageJ software (NIH, Ver. 1.4, USA). At least 10 randomly selected high-power fields (HPFs) were selected for the evaluation of fibrotic changes.

####  Immunohistochemistry (IHC) Staining

 The possible angiogenesis effects of YKL-40 and YKL-40@AFExos on infarcted myocardium were monitored by the local density of vWF ^+^ and α-SMA ^+^ vessels at the border zone ^29^. In short, paraffin-embedded sections were deparaffinized and rehydrated according to the standard protocols. Samples were incubated with citrate buffer to retrieve immunogenic antigens, followed by treatment with 3% H_2_O_2 _to neutralize the endogenous peroxidase activity. To reduce non-specific binding sites, slides were incubated with 2% BSA solution for 30 minutes and anti-α-SMA (Cat no: IS70030-2: Dako; Denmark) and anti-vWF (Cat no: GA067-61-2: Dako; USA) overnight at 4°C. Slides were washed several times with PBS solution, and the immunoreactive sites were detected using HRP-conjugated secondary antibodies (EnVision + Dual Link System HRP kit; Dako, Denmark) and DAB solution. The number of vWF ^+^ and α-SMA ^+^ vessels was monitored in at least 10 HPFs related to border zones and compared with different experimental groups.

###  Biochemical Analysis

 The serum levels of lactate dehydrogenase (LDH; Cat No. 613036, Pars Peyvand Co, Iran), aspartate aminotransferase (AST; Cat No. 613033, Pars Peyvand Co, Iran), and alanine aminotransferase (ALT; Cat No. 613032, Pars Peyvand Co, Iran) were measured after two weeks at the time of euthanasia. To this end, a 500-1000 µl blood sample was sampled from each rat heart, and the serum was isolated using centrifugation (3000 rpm for 15 minutes). The systemic content of LDH, AST, and ALT was measured and compared to that of the control rats.

###  Statistical Analysis 

 Data [mean ± SD] were analyzed using one-way ANOVA followed by Tukey’s post hoc test in GraphPad Prism software (Ver. 8.4.3; CA, USA). P values < 0.05 were considered statistically significant. To reduce conscious and unconscious bias, the analyses, histological examination, and interpretation were done blindly.

## Results

###  AFExo Characterization and Immunophenotyping

 In the present study, AFExos were used as biocarriers for the delivery of YKL-40 into the ischemic myocardium. The physicochemical properties of AFExos isolated using ultracentrifugation were studied using various techniques (Figure 2A-E). SEM and TEM imaging revealed that the isolated AFExos are spherical and encompass relatively heterogeneous particles in terms of mean size (Figure 2A-B). The analysis of AFExo sizes using the SEM images via ImageJ software indicated a mean geometric diameter of 82.3 ± 21.8 nm, while DLS analysis showed a larger hydrodynamic diameter of 189.9 ± 27.1 nm (Figure 2C-D). Based on our data, a mean zeta potential values of 17.7 ± 3.5 mV were achieved in isolated AFExos. Of note, the PDI values in the DLS analysis were at range between 0.279-0.312, indicating relatively homogenous size distribution. Using western blotting, the existence of tetraspanins such as CD63 and CD81, along with TSG101 belonging to the endosomal sorting complex required for transport (ESCERT) complex, was indicated in the lysates of AFExos (Figure 2E). These data confirmed the typical Exo morphology and phenotypes using the current isolation protocol.

**Figure 2:**
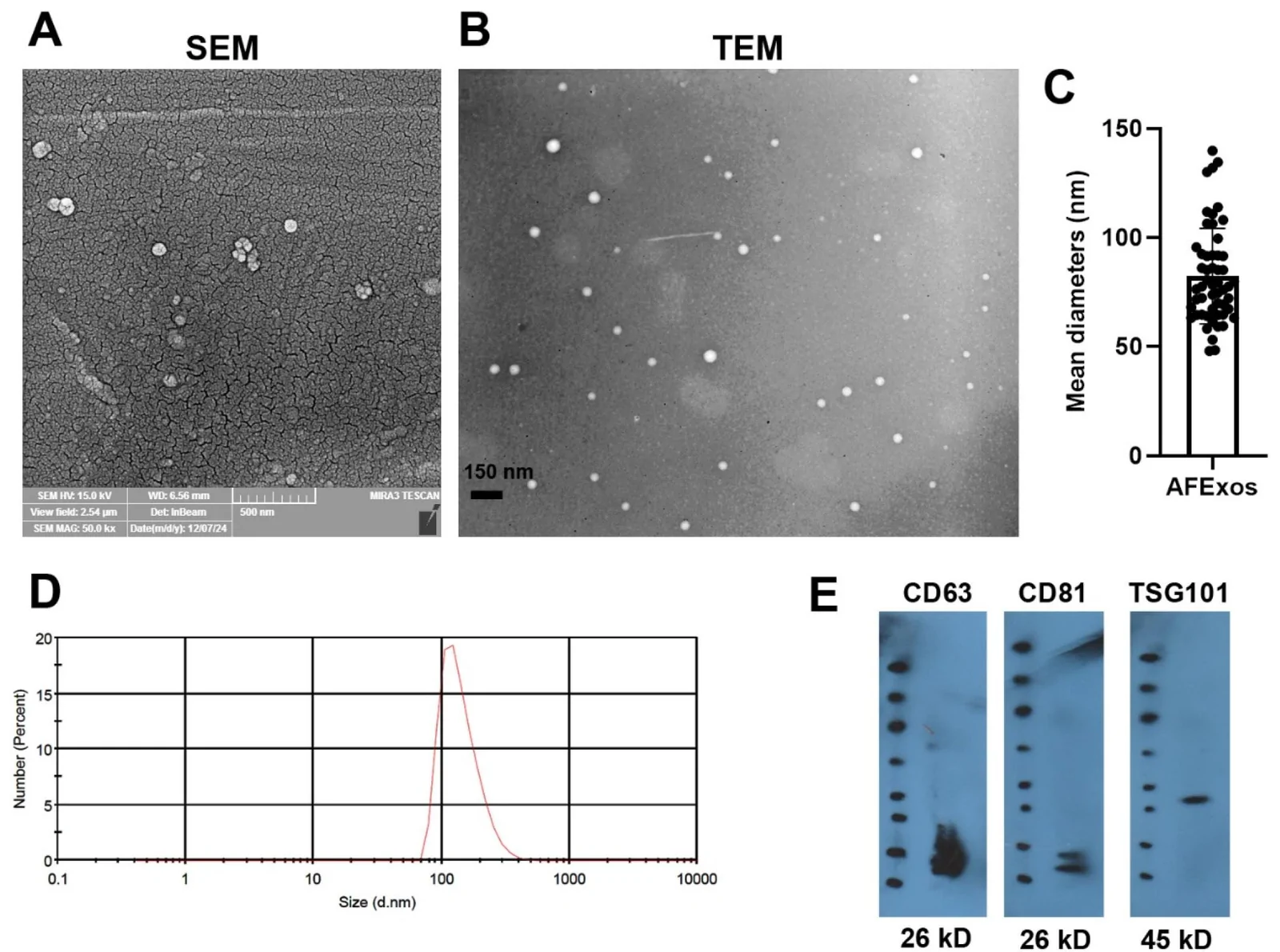
Characterization and immunophenotyping of isolated AFExos using ultracentrifugation (**A**-**E**). SEM and TEM images indicated spherical morphologies and a heterogeneous exosomal population (**A** and **B**). AFExo mean diameter was calculated using ImageJ software (**C**). DLS analysis revealed a hydrodynamic size of 189.9 ± 27.1 nm with a zeta potential value of 17.7 ± 3.5 mV (**D**; n = 3). Western blotting of AFExo lysates for the detection of tetraspanins (CD63 and CD81), and ESCRT component TSG101 (**E**; data were obtained from three pooled samples)

###  Peptide Loading 

 In the present study, the sonication approach was used for the loading of YKL-40 peptide onto the AFExos. To confirm the peptide loading, the existence of YKL-40 peptide was monitored in sonicated AFExos using western blotting. Data indicated basal levels of YKL-40 in AFExos released inside the amniotic fluid (Figure 3A-B). Sonication led to a ~6.1-fold increase of YKL-40 onto the AFExos compared to the naïve counterparts (Figure 3B; *P* < 0.01). Besides, the levels of supernatant YKL-40 peptide were measured using the BCA assay pre- and post-sonication (Figure 3C). Based on our calculation, the loading efficiency of YKL-40 onto AFExos was about 55.5 ± 1.5%.

**Figure 3:**
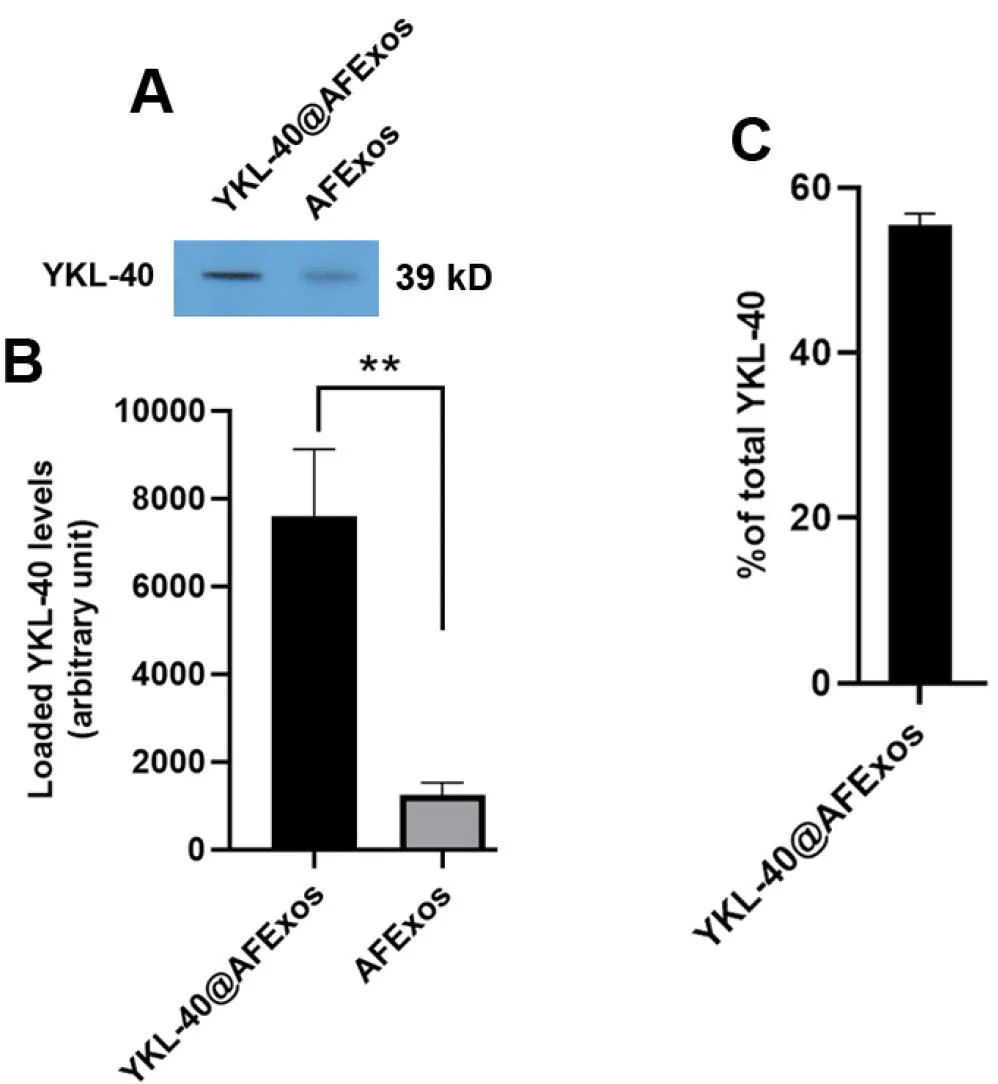
The loaded YKL-40 peptide onto AFExos was measured using western blotting (**A** and **B**; data were obtained from three pooled samples) and BCA assay (**C**; n = 3). Western blotting confirmed the existence of YKL-40 peptide in AFExos lysates when compared to naïve AFExo counterparts (**A** and **B**). BCA assay indicated loading efficiency of 55.5 ± 1.5% using the sonication method. Student t-test. **P < 0.01

###  YKL-40@Exos Efficiently Reduced the Infarct Area

 To confirm the induction of MI in a rat model, permanent LAD ligation was done using an open-chest surgical approach according to our previously conducted study (Figure 4A-B).^29^ Data indicated that LAD caused visible cyanosis in the cardiac tissue due to ischemic changes about 20-30 minutes post-LAD ligation (Figure 4A). Along with these changes, in the electrocardiogram, elevation of the ST segment was evident with the progression of ischemic changes (Figure 4B). Masson’s trichrome staining indicated that the LAD ligation can lead to the progression of blue-colored collagen fiber into the myocardial parenchyma in the MI group as compared to the healthy control heart tissue (Figure 5A-B). Of note, it was shown that AFExos alone can promote anti-fibrotic effects via the reduction of collagen fiber deposition in the ischemic area in infarcted rats. Besides, the direct administration of YKL-40 alone led to the formation of aligned collagen fibers surrounding cardiomyocytes (Figure 5A). Compared to the MI, AFExos, and YKL-40 groups, the administration of YKL-40@AFExos led to a profound reduction of dense collagen fibers; the least collagen content was evident in this group. The calculation also indicated the significant reduction of infarct size/each slide in YKL-40@AFExos compared to the YKL-40 and MI groups (*P* < 0.05; Figure 5B). Notwithstanding, the greatest infarct volume was achieved in the MI groups after the ligation of LAD. Despite a reduction of the infarct zone in AFExos and YKL-40 groups, these values did not reach a statistically significant difference compared to the MI groups. Therefore, these data show the potent anti-fibrotic properties of YKL-40@AFExos in MI rats with a profound reduction of infarct size. The injection of AFExos exhibited superior anti-fibrotic properties compared to the YKL-40 factor. General histological examination was also used to evaluate the cardiac tissue remodeling after the induction of MI and administration of free AFExos, YKL-40, and YKL-40@AFExos (Figure 5C). H & E staining showed the existence of red/pink colored wavy collagen fibers with numerous blue cell nuclei, indicating the progression of fibrotic change two weeks after LAD ligation. Notably, the injection of AFExos led to the recruitment of numerous immune cells within the ischemic area, coinciding with concomitant reduction of collagen fibers, indicating the activation of reparative mechanisms to support injured cardiomyocytes (Figure 5C). In the YKL-40 group, fibrotic areas with juxtaposed vascular beds are detected, while the load of YKL-40 onto AFExos contributed to the protection of relatively large numbers of cardiomyocytes, with the reduction of recruited immune cells. These features are consistent with the fact that the injection of YKL-40@AFExos into the ischemic myocardium can protect against the extension of fibrotic changes and cardiomyocyte death via the regulation of immune cell homing and collagen fiber deposition.

**Figure 4:**
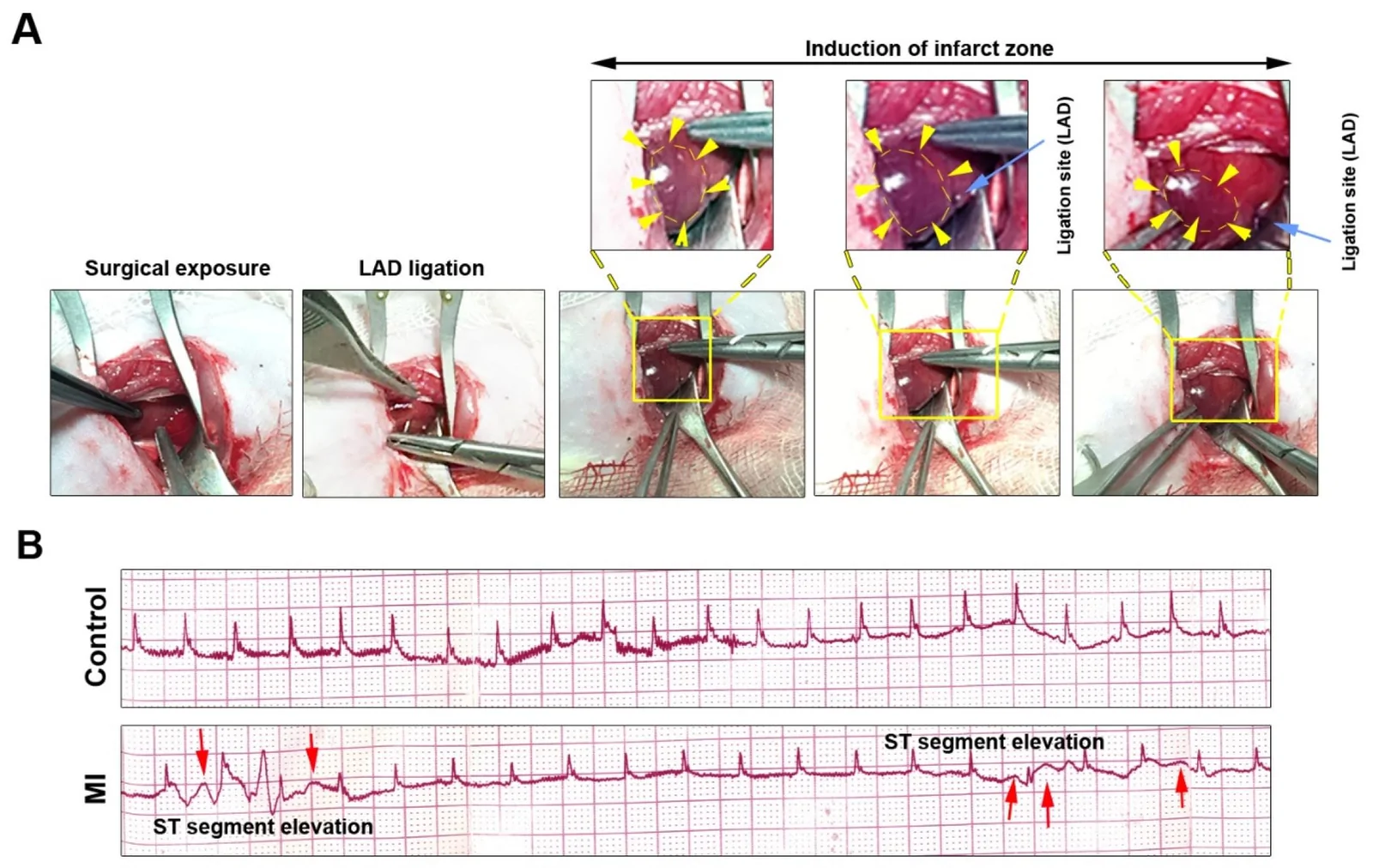
The induction of MI was done in rats using left thoracotomy and LAD ligation. The interruption of blood perfusion into the anterior surface of the left ventricle led to ischemia (**A**; yellow arrowheads). Electrocardiogram profile indicated ST-segment elevation in MI rats compared to the control group (**B**; red arrows).^29^ Copyright 2024. International Journal of Biological Macromolecules

**Figure 5:**
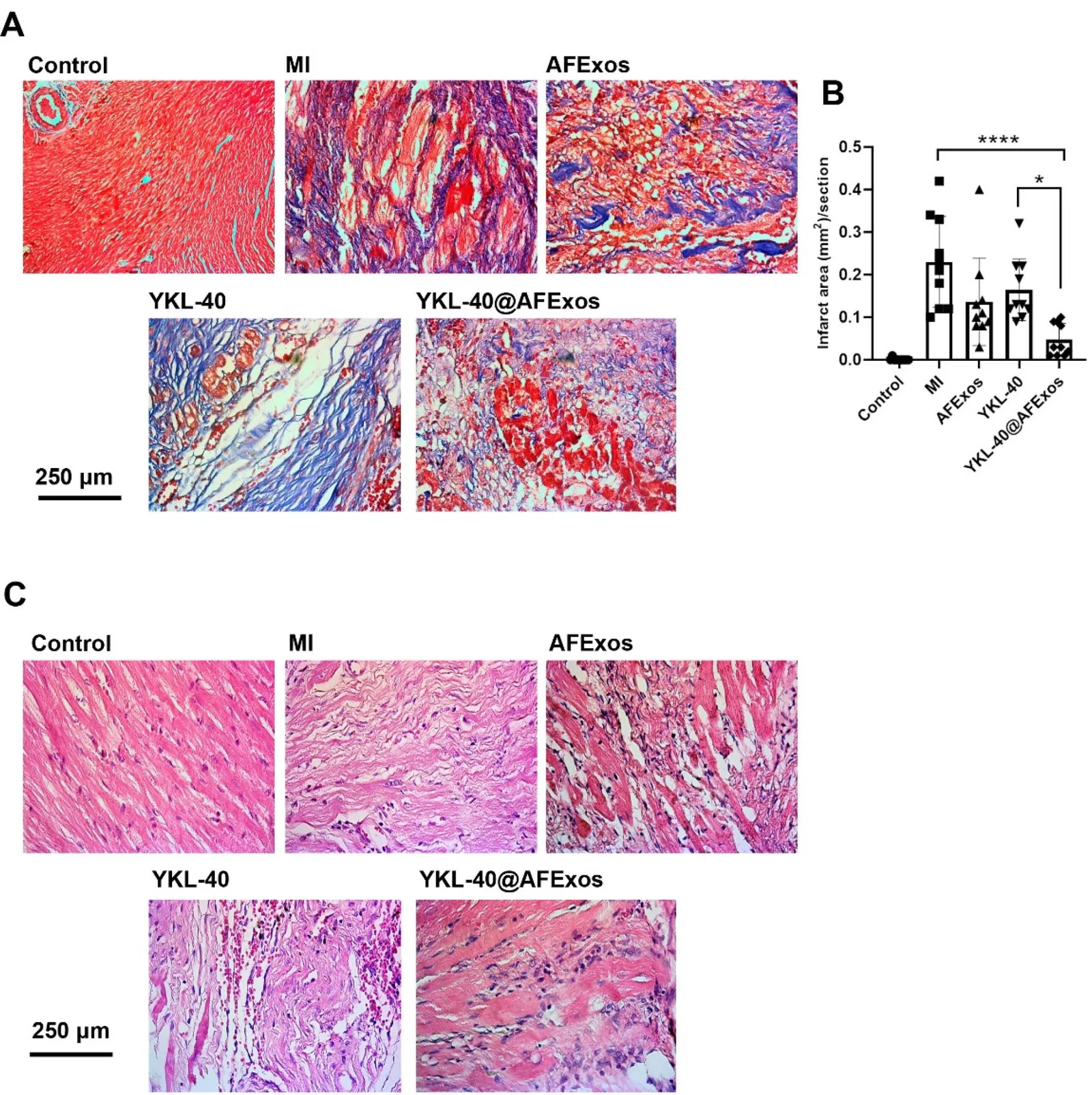
Monitoring fibrotic changes and infarcted area using Masson’s trichrome staining (**A-B**). Data showed the reduction of fibrotic changes and infarcted area in rats that received YKL-40@AFExos (*P* < 0.05). H & E staining (**C**). Data showed massive collagen fiber deposition and loss of cardiomyocytes in MI rats. The injection of AFExos and YKL-40@AFExos, but not YKL-40, led to the reduction of collagen fiber deposition and increased cardiomyocyte survival. One-Way ANOVA with Tukey post hoc analysis (n = 10). *P < 0.05; and ****P < 0.0001

###  YKL-40@Exos Stimulated Angiogenesis in Infarcted Rats

 Using IHC staining, the density of vWF ^+^ and α-SMA ^+^ vascular units was monitored in different experimental groups after two weeks (Figure 6A-D). It was found that the induction of LAD ligation led to the density of numerous α-SMA ^+^ myofibroblasts, which are responsible for myocardial scar. In line with these findings, α-SMA ^+^ arterioles were lowest in MI rats, indicating the generation of an abnormal fibrotic area two weeks after induction of myocardial ischemia (Figure 6A-B). The injection of AFExos and YKL-40 can separately increase the number of α-SMA ^+^ arterioles compared to the MI groups (*P* < 0.05). Interestingly, the highest level of arteriole formation was achieved after administration of YKL-40@AFExos compared to the AFExos and MI groups. Despite the increase of α-SMA ^+^ arterioles in YKL-40, the existence of α-SMA ^+^ myofibroblasts highlights simultaneous scar formation, like the MI group. Besides, a similar pattern was found in the generation of vWF ^+^ capillaries in MI rats after the administration of AFExos, YKL-40, and YKL-40@AFExos. To be specific, YKL-40@AFExos transplantation can increase local capillary density compared to AFExos, YKL-40, and MI groups (*P* < 0.05). It seems that injection of AFExos, and/or YKL-40, stimulated the formation of vWF ^+^ compared to the control MI rats. These data showed the superior angiogenesis properties of YKL-40@AFExos in MI rats, especially when compared to the AFExos and YKL-40 groups.

**Figure 6:**
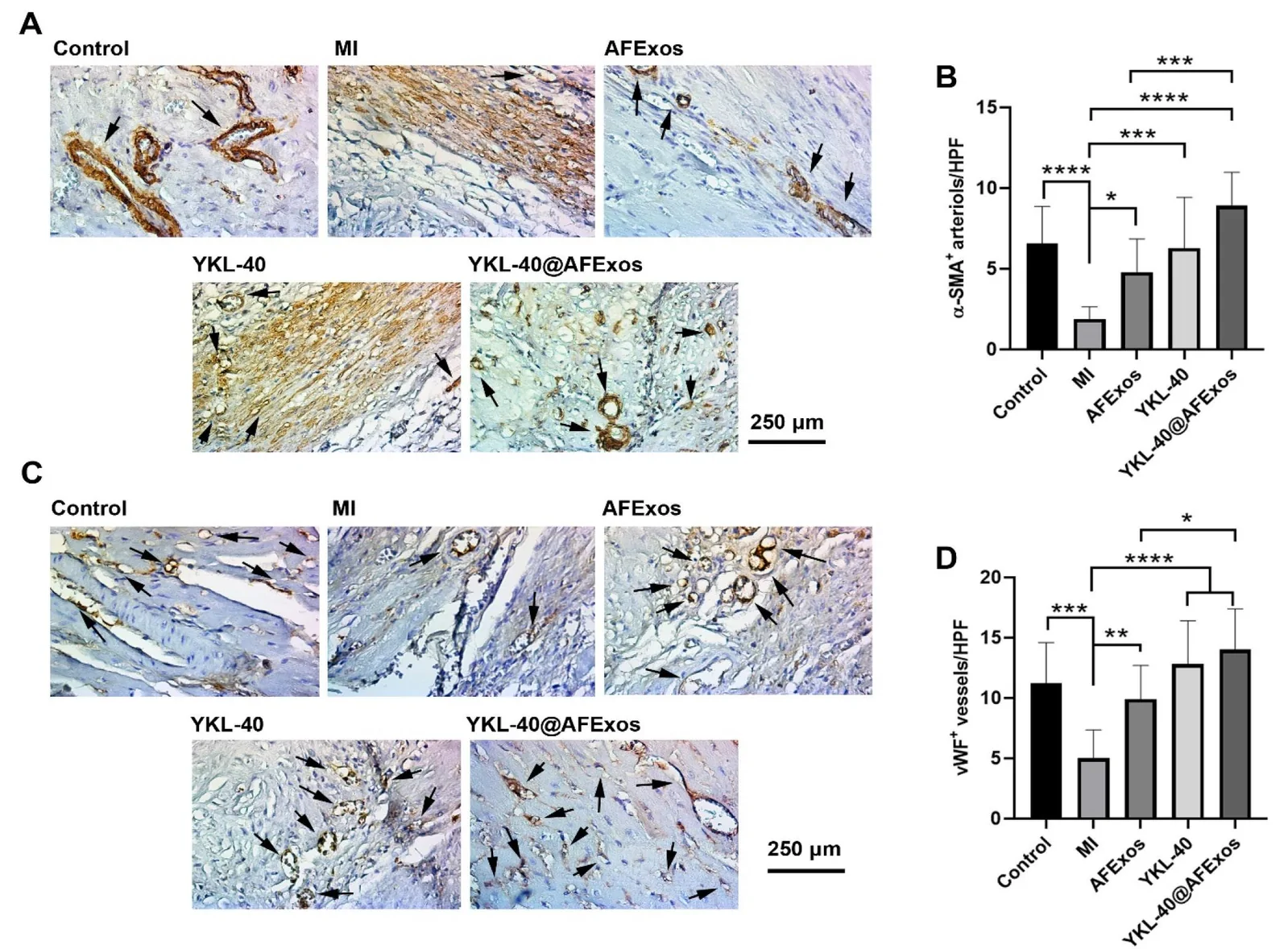
IHC staining of myocardium in MI rats two weeks after the administration of AFExos, YKL-40, and YKL-40@AFExos. Data showed the superior potential of YKL-40@AFExos in the induction of angiogenesis via the formation of α-SMA ^+^ and vWF ^+^ vessels in MI rats. One-Way ANOVA with Tukey post hoc analysis (n = 10). *P < 0.05; **P < 0.01; ***P < 0.001; and ****P < 0.0001

###  YKL-40@AFExos Stimulate Angiogenesis Factors in Infarcted Myocardium 

 Protein levels related to angiogenesis (VEGF, VEGFR-1, and -2), autophagy (LC3), and apoptosis (Bad and Bax) were monitored using western blotting (Figure 7). Data indicated that the levels of apoptotic factor Bad were significantly increased as compared to the control group (*P* < 0.05; Figure 7). Treatment of MI rats with AFExos, YKL-40, and YKL-40@AFExos reduced the Bad levels inside the infarcted myocardium and reached near-basal levels, in which non-significant differences were achieved in terms of Bad levels compared to control rats (*P* > 0.05; Figure 7). Despite the increase in Bax levels in MI rats, these values did not reach a statistically significant level compared to the control rats (*P* > 0.05; Figure 7). Notably, the injection of YKL-40 and YKL-40@AFExos led to a significant reduction of Bax inside infarcted myocardium compared to the MI rats (*P* < 0.05). We also found that the induction of ischemia in rat myocardium led to the activation of autophagic response (increased LC3-II/I ratio) related to the control rats (*P* < 0.01; Figure 7). It seems that the administration of AFExos did not alter the abnormal autophagic response when compared to control groups. Despite the slight reduction of the LC3-II/I ratio in AFExos rats, these levels remained statistically significant (*P* < 0.01; Figure 7). Of note, a similar trend was achieved in terms of autophagic response and autophagy after the transplantation of YKL-40 and YKL-40@AFExos, in which the LC3-II/I ratio was significantly reduced when compared to the MI group (*P* < 0.05; Figure 7). It was found that the combination of YKL-40 and AFExos exerted higher anti-autophagic properties related to the YKL-40 factor. These data indicate that either transplantation of YKL-40 or YKL-40@Exos can blunt the autophagic response activated after the induction of ischemic change in rat myocardium. It was also found that the induction of MI led to the suppression of the VEGF factor as compared to the control rats (*P* < 0.05; Figure 7). The injection of YKL-40 and YKL-40@AFExos led to an increase of VEGF near control levels, in which statistically non-significant differences were obtained compared to the control group. Interestingly, the injection of AFExos did not increase the VEGF, and an MI-like condition was recorded in terms of VEGF. We also found statistically non-significant differences in VEGFR-2 levels in all experimental groups compared to the control group (*P* > 0.05; Figure 7). The induction of MI contributed to the significant reduction of VEGFR-1 in comparison with the control rats. Again, transplantation of YKL-40 and YKL-40@AFExos caused a local increase of VEGFR-1; however, these values were significant in the YKL-40 group (*P* < 0.05; Figure 7). The injection of AFExos alone did not alter the MI-induced reduction of VEGFR-1, in which significant suppression of VEGFR-1 was evident between the AFExos and control groups. Taken together, these data indicate that either YKL-40 or YKL-40@AFExos has superior potential to control the progression of autophagy and apoptosis after ischemic changes in myocardium. In terms of angiogenesis factor, it should be noted that YKL-40 and YKL-40@AFExos exert superior angiogenesis properties compared to AFExos after the MI induction.

**Figure 7:**
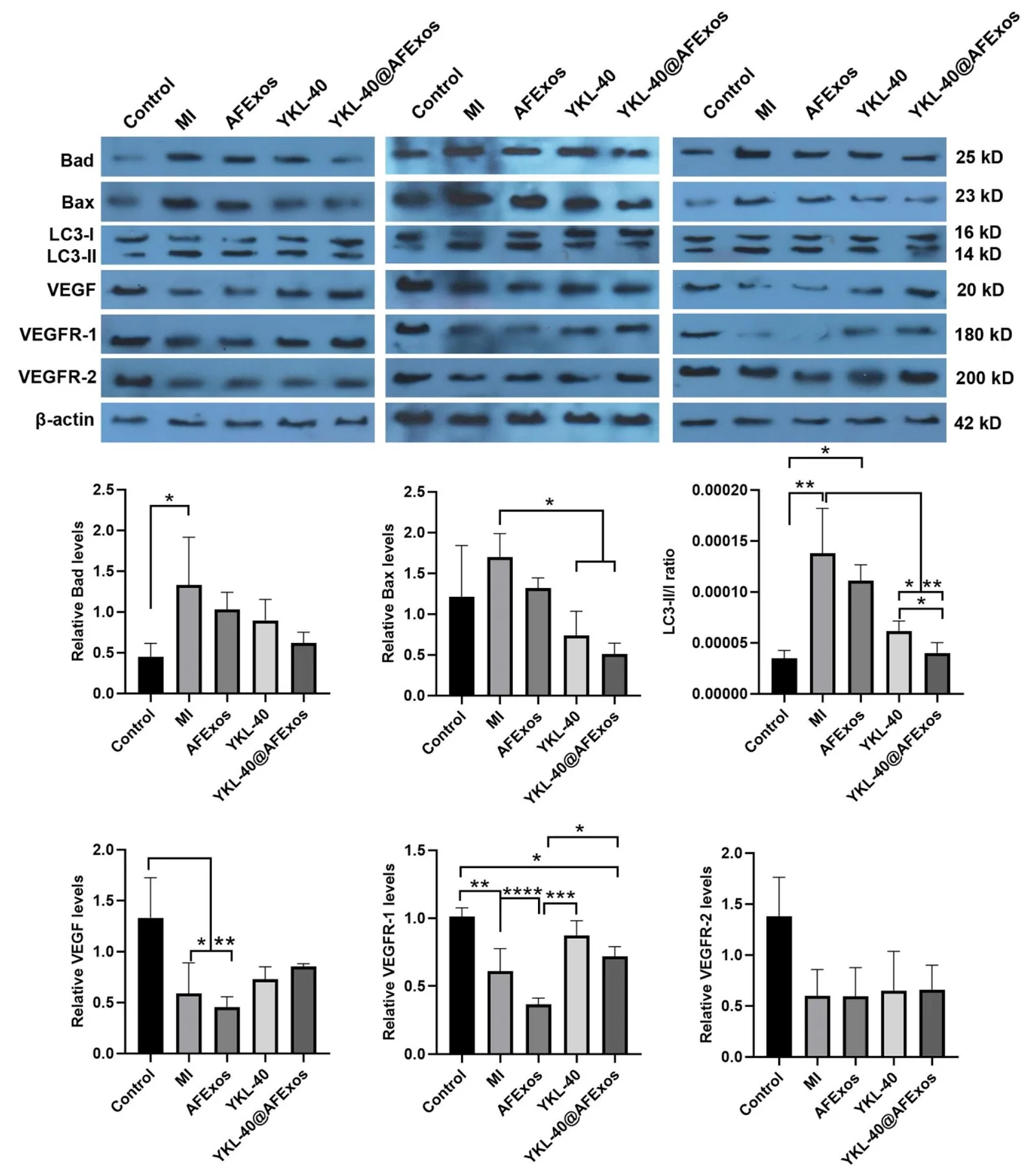
Western blotting for monitoring protein levels of apoptosis (Bad, and Bax), autophagy (LC3), and angiogenesis (VEGF, VEGFR-1, -2) two weeks after injection into infarcted myocardium. One-way ANOVA with Tukey post hoc analysis (n = 3). Data were collected from six rats and pooled into three rats. *P < 0.05; **P < 0.01; ***P < 0.001; and ****P < 0.0001

###  Biochemical Profile

 To monitor the possible cardiomyocyte injury, the systemic levels of enzymes such as AST, ALT, and LDH were measured two weeks after the transplantation of AFExos, YKL-40, and/or YKL-40@AFExos (Figure 8). Data indicated the lack of statistically significant differences in systemic levels of LDH, AST, and ALT in different experimental groups after 14 days compared to the control rats (*P* < 0.05). These data indicated that neither naïve AFExos nor YKL-40 nor YKL-40@AFExos exhibited direct benefits in the levels of the above-mentioned enzymes.

**Figure 8:**
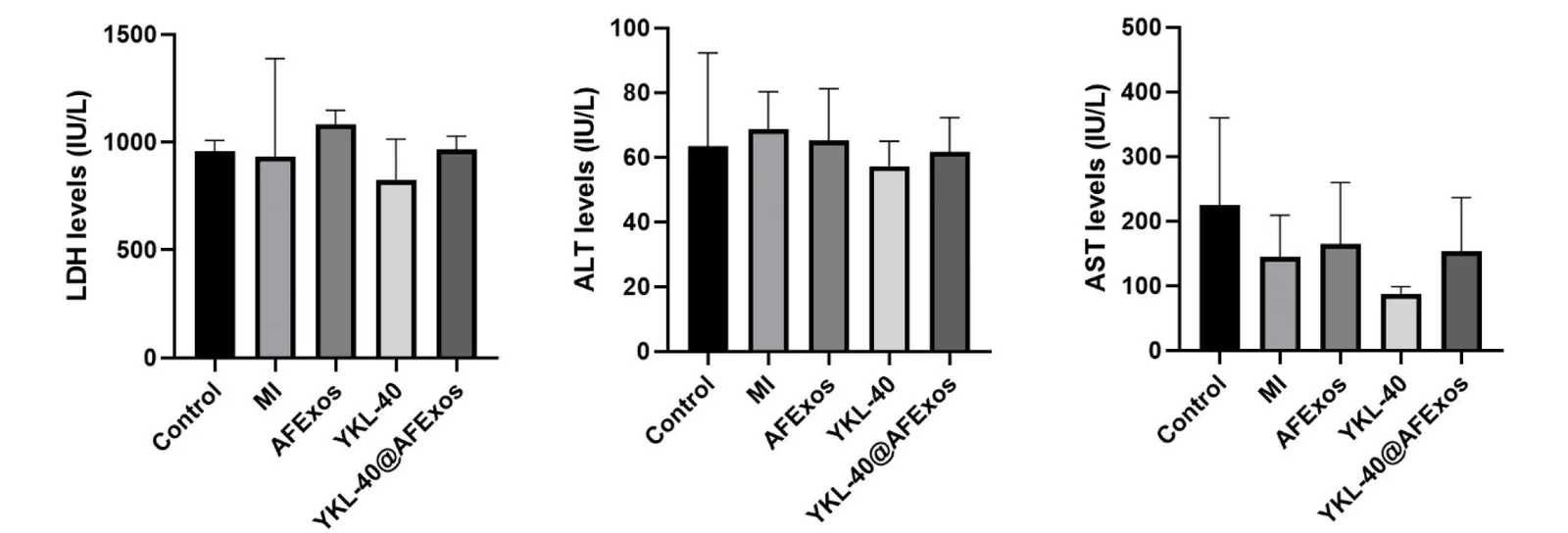
Biochemical analysis of LDH, AST, and ALT enzymes two weeks after transplantation of AFExos, YKL-40, and YKL-40@AFExos in infarcted rats (n = 3). One-way ANOVA with Tukey post hoc analysis. Data were collected from six rats and pooled into three rats

## Discussion

 During recent years, several preclinical studies have been done to monitor the eligibility of Exos for increasing delivery efficiency and retention time in the target tissues.^30,31^ It is thought that Exos are natural and tiny nanoparticles with the potential to cross several biological barriers with minimal toxicity.^32^ These features make Exos promising delivery platforms for several therapeutic purposes, especially the induction of angiogenesis and vascularization during ischemic changes.^15^ Here, AFExos were used as a delivery platform for YKL-40 into the infarct region. It was suggested that amniotic fluid is an acceptable EV-rich biofluid for regenerative purposes.^33^ These EVs originate from the fetoplacental unit with a high density of growth factors, proteins, and immunoglobulins, which support the development and growth of the fetus.^34^

 Multiple studies have revealed that YKL-40 is a fibrosis promoter and increases after the onset of several pathological conditions.^35^ For example, this factor is altered in individuals with severe liver fibrosis,^36,37^ idiopathic pulmonary fibrosis,^38^ etc. Regarding the fact that control of fibrosis and induction of angiogenesis are critical items in the regeneration of cardiac tissue post-ischemic changes, and long-term heart function. These phenomena should be precisely controlled to inhibit the formation of stable scar tissue. Of note, studies have indicated the capacity of inflamed cells and tumor cells to release YKL-40 for stimulating the vascular bed formation.^39^ Besides its role in the increase of blood perfusion into the damaged area, some data confirm the critical impact of YKL-40 in the progression of cardiovascular dysfunction and coronary artery disease.^40^ Along with these descriptions, there is a discrepancy in the function and role of YKL-40 in the biological system, especially in MI. Therefore, we aimed to assess whether the fibrotic properties or angiogenesis of free YKL-40 or YKL-40@AFExos is the dominant biological effect in infarcted rats after two weeks.

 We found that intact AFExos harbor small levels of YKL-40 peptide isolated using the ultracentrifugation method. Besides, the control and analysis of supernatants in sonicated samples revealed that nearly 50% of soluble YKL-40 factor was loaded onto the AFExos. Western blotting confirmed about a 6.1-fold increase of YKL-40 peptide in exosomal lysates, indicating the efficiency of the current protocol to load the target molecules onto the AFExos. Because amniotic fluid encompasses a heterogeneous Exo population, it is logical to hypothesize that this factor can originate from various cell lineages.^41^ Although it should not be forgotten that the increase of this factor is related to several inflammatory conditions and pathologies.^35^ The physiological YKL-40 levels are involved in migration and tissue regeneration in certain cell lineages, such as ECs and fibroblasts.^35^ Commensurate with these descriptions, one can hypothesize that the basal levels of YKL-40 in biofluids, even inside AFExos, correlate with the regulation of fetal growth and developmental progression.

 It has been thought that mild sonication cannot alter the integrity of Exos as used in the present study.^42^ The sonication step was done according to a previously established protocol by our research group. Based on our data, the induction of MI led to accumulated blue-colored collagen fibers in the MI group after two weeks, as compared to the controls, indicating successful LAD ligation and ischemic induction in a rat model. Thus, the deposition of collagen fibers is closely associated with aberrant remodeling and restricted blood flow, promoting cardiomyocyte death after being exposed to ischemia.^43^ We also found that administration of naïve AFExos can reduce the collagen fiber deposition in the infarcted zone, leading to a mitigation of the massive myocardial remodeling and reduction of the infarcted area. However, these features were not statistically significant compared to the matched MI group. It has been found that AFExos can exert anti-fibrotic properties in various pathological conditions via the reduction and regulation of SMAD proteins belonging to the TGF-β signaling pathway.^44,45^ The lack of significant differences in terms of fibrosis rate in AFExos rats can be related to several factors. Various parameters, such as inconsistencies in cargo type, administration route, and dose, can alter the therapeutic efficiency of Exos.^46^ Here, a single AFExos dose was used for the alleviation of MI-related pathologies in rats, which may not be sufficient to attenuate the fibrosis and promote the expansion of the necrotic area. Notably, short half-life and retention time are major challenging issues in the application of Exos in the context of cardiac tissue regeneration.^47^ Thus, it is recommended that the dosage, dosing intervals, and suitable administration route should be adequately validated for the MI conditions. More interestingly, the administration of YKL-40 led to an increase in local collagen fibers at the site of injury. Several studies have found a close relationship between the local increase of YKL-40 and the progression of fibrotic changes in certain organs.^48^ The expression of this factor following inflammatory conditions contributes to the proliferation of fibroblasts, and direct binding to type I, II, and III collagen fibers, leading to the formation of scar tissue and reducing the collagenolytic activities.^49^ Based on our data, the administration of YKL-40@AFExos can significantly reduce scar tissue formation after MI induction. One reason for the anti-fibrotic properties of loaded YKL-40 could be related to the fact that excessive secretion and sudden release of YKL-40 into the ischemic area can exacerbate the pathological condition and fibrotic changes via activation of FAK and MAPK signaling pathways.^50^ Jing-Lun and co-workers found that overexpression of YKL-40 in mice with hepatic injury intensifies the pathological conditions via the activation of the TF-PAR1 pathway, and local increase of chemokine ligand 2 and IP-10.^51^ Of course, it should not be forgotten that the existence of different genetic factors, peptides, and cytokines can also exert anti-fibrotic properties simultaneously. For example, let-7-5p, miR-22-3p, miR-27a-3p, and miR-21-5p were indicated in AFExos with the potential to inhibit type I and II TGF-β receptors, resulting in the reduction of myofibroblast differentiation.^52^ It is believed that the physiological levels of YKL-40 promote tissue regeneration and remodeling via the regulation of fibroblast function.^35^ It is thought that the direct injection can lead to hyperactivation of local fibroblasts via the MAPK signaling axis at the site of injection and scar tissue formation.^53^ Therefore, it seems that AFExos can release the YKL-40 cargo into the infarcted myocardium and sustain over time compared to the rats that received direct YKL-40 injection. H & E staining revealed the existence of wavy collagen fibers in the MI groups. Unlike the MI group, numerous immune cells with healthy and necrotic cardiomyocytes can be detected after administration of AFExos, indicating active remodeling tissue. Similar to the MI group, scar tissue with blood vessels is evident in the YKL-40 group. In the YKL-40@AFExos group, the number of immune cells were reduced with higher density of cardiomyocyte per microscopic field. Previously, it was suggested that AFExos can enhance neonatal mouse cardiomyocyte cycle progression via the Yap signaling pathway and F-actin polymerization.^54^ Therefore, we hypothesize that the direct cardiogenic properties of AFExos are related to exosomal cargo rather than the direct effect of YKL-40 in injured cardiomyocytes in the border zone. IHC analysis revealed the higher angiogenic properties (CD31 ^+^ capillaries and α-SMA ^+^ arterioles) of YKL-40@AFExos compared to YKL-40 and AFExos groups. Interestingly, the arteriogenesis properties of YKL-40 coincide with a concomitant increase of α-SMA ^+^ fibroblasts at the site of infarction as indicated by Masson’s trichrome staining. Thus, we indicated that free YKL-40 can promote the proliferation of local fibroblasts two weeks after MI induction, while loaded YKL-40 inside the AFExos can stimulate angiogenesis with minimal fibrotic changes. It seems that the angiogenic properties of AFExos are associated with the induction of HIF-1α and the VEGF signaling pathway, leading to the generation of *de novo* blood vessels.^55^ Along with these features, encapsulated YKL-40 inside the AFExos can, in turn, stimulate the generation of new vascular units. Therefore, the activation of angiogenesis in rats receiving YKL-40@AFExos is presumably associated with the simultaneous activity of loaded YKL-40 and endogenous AFExo cargo. It is suggested that the increase of local CD31 and α-SMA ^+^ blood vessels in the YKL-40 group can be closely related to the recruitment of immune cells and inflammation-associated angiogenesis, such as IL-13, IL-6, TNF, IL-13, and IL-18 etc.^35,56,57^ We found that protein levels of Bad, Bad, and LC3-II/-I ratio were increased following the induction of ischemic changes within the myocardium, and administration of free YKL-40 peptide and YKL-40@AFExos can reduce the apoptotic cardiomyocytes and blunt the autophagic response in the MI rats. To be specific, it can be said that AFExos harboring YKL-40, or free YKL-40, can lead to the reduction of excessive autophagy response and inhibition of apoptotic cell death. It is hypothesized that these effects in the YKL-40 group can be associated with increased angiogenesis properties. Whether the inhibition of autophagy response in MI rats after direct injection of YKL-40 is pathological or can help to restore the cardiac cell function should be addressed by future studies. The lack of significant changes in protein levels of VEGF and VEGFR-2 in the YKL-40 group can be related to the fact that these factors can engage certain angiogenesis signaling pathways, such as VEGFR-1 signaling pathways, inside the ischemic region. In contrast to VEGF and VEGFR-2, the significant increase of VEGFR-1 in YKL-40 and YKL-40@AFExos groups compared to MI and AFExos may be associated with the negative role of VEGFR-1 on VEGFR-2. Both receptors are engaged and stimulated in parallel in the presence of VEGF, and VEGFR-2 is the main receptor to control the proliferation and angiogenic potential of ECs. Previous data have confirmed that VEGFR-1 acts as a decoy for VEGFR-2 to control and regulate angiogenesis outcome via the sequestration of excessive VEGF contents in the target tissues.^58^ It should not be neglected that the time of study can influence the intensity, frequency, and sequence of cells’ bioactivity in the healing tissues.^59^ To be honest, conflicting and inconsistent findings across numerous experiments related to the exact anti- or pro-angiogenesis properties of YKL-40 under pathological conditions are a common challenge.^60,61^ It was also noted that systemic levels of ALT, AST, and LDH did not reach statistically significant levels in different experimental groups compared to the MI rats. One reason would be that the significant changes in systemic levels of these enzymes can be monitored just a few days after the induction of MI, and the replacement of injured cardiomyocytes with fibroblasts or myofibroblasts can contribute to the lack of significant changes in blood enzymes such as ALT, AST, and LDH.^29^

 The current study acknowledges several limitations and bottlenecks that need further experiments. Along with YKL-40, it is mandatory to measure protein levels of angiogenesis factors inside the AFExos to address the precise angiogenesis properties of these particles alone or in combination with loaded YKL-40 peptide. In the current experiment, a full-length peptide was used for loading onto the AFExos. Conduction of further analyses, such as a floating assay, can help to confirm suitable loading properties. Monitoring the retention time, distribution, bioactivity, and stability of free YKL-40 and YKL-40@AFExos is crucial for understanding their behavior within the ischemic myocardium and the angiogenesis properties. It is suggested that future studies use multiple doses of free or loaded YKL-40 to precisely address the delivery-related effects. Considering a pleiotropic role of YKL-40 peptide in different pathological conditions, the impact of dosage, administration interval, and prolonged effects can help understand YKL-40 angiogenesis properties under ischemic conditions. It is suggested that future studies assess the possible simultaneous positive and negative effects of immune cells and inflammatory response on vascularization potential driven by YKL-40. Here, the direct interaction of YKL-40 with VEGFRs was not precisely determined. The existence of possible simultaneous anti-angiogenesis inhibitors, such as thrombospondin-1, and receptor activity, such as CD47, should be addressed by future studies. To monitor the cardiac output, function, geometry, and blood flow, Doppler echocardiography is suggested for future studies along with other molecular and histological examinations. The proangiogenic and fibrotic properties of YKL-40 in loaded and free forms should be addressed by further studies. Whether and how the YKL-40 delivery forms predetermine the particular regenerative outcomes should be precisely investigated. The two-week follow-up period was used in the present experiment to monitor the restoration of ischemic cardiac tissue. Regarding the fact that the healing process is an intricate mechanism and regenerating cells need more time to achieve appropriate integration and functional maturation, it is logical to conduct numerous studies to circumvent these pitfalls. Translation of current data into human medicine has several limitations. The correct dose, injection interval, and treatment schedules should be exactly determined in human counterparts using some clinical trials. The reliable Exo sources for the load of target cytokines and molecules with minimal side effects and immune rejection are at the center of attention in this regard.

## Conclusion

 In conclusion, it was shown that direct injection of the YKL-40 peptide in a rat model of acute MI led to simultaneous fibrotic changes and induction of angiogenesis. The administration of YKL-40@AFExos promotes simultaneous angiogenesis response and cardiac tissue regeneration. Based on the present data, AFExos are valid bioshuttles in the delivery and introduction of YKL-40 into the ischemic regions. More data are mandatory to assess the possible effect of loaded YKL-40 on cardiac tissue function and differentiation capacity of various cell lineages inside the myocardium following acute infarction.

## Competing Interests

 None declared.

## Consent for Publication

 Not applicable.

## Ethical Approval

 The study was registered as titled “Assessment of the Angiogenic Effects of Exosomes Derived from Amniotic Fluid Transfected with YKL-40 Factor in the Model of Rat Myocardial Infarction”. All steps of this study were approved by the Elite Researcher Grant Committee under the ethical code of [IR.NIMAD.REC.1397.512] from the National Institute for Medical Research Development (NIMAD), Tehran, Iran, on 2019-02-02, and the Ethical Committee of Tabriz University of Medical Sciences under the ethical code of IR.TBZMED.VCR.REC.1397.395 on 2019-01-28. Animals were treated according to the previously published ARRIVE guidelines.
